# Interdiciplinares impactanto actions on prevention of ventilator-associated pneumonia in patients of trauma intensive care unit

**DOI:** 10.1186/2047-2994-4-S1-P244

**Published:** 2015-06-16

**Authors:** A Sola

**Affiliations:** 1 Controle de infecção, Hospital 9 de Julho, São Paulo, Brazil

## Introduction

Ventilator associated pneumonia is the most common among hospital infections, especially in intensive care unit patients Trauma. With the concern to improve the quality provided in assistance to patients on mechanical ventilation, intensive care created a multidisciplinary group to develop, evaluate and implement the necessary measures to prevent ventilator-associated pneumonia.

## Objectives

Reduce the incidence of ventilator-associated pneumonia in the intensive care unit of trauma.

## Methods

This is a quantitative prospective study, conducted from January 2010 to December 2014 in a 10-bed intensive care unit of trauma high complexity hospital, at Sao Paulo, Brazil. In 2009 we implemented the multidisciplinary group: supervision of nursing, medical coordinator, intensivist and physiotherapist intensive care, pharmacy, nutrition and infection control. The adopted actions taken over the years were: Weekly discussion of cases with evaluation of the clinical condition of the patient, sedation interruption and weaning; protocol review as oral hygiene, threshold elevation; Review of materials and respiratory care equipment.

## Results

The year 2009 was the beginning of VAP prevention actions of the multidisciplinary group, with only 4 reviews, and no case of infection. In 2010, the first year of follow up were 785 assessments with 25.85% of the membership shares and 0 (zero) EPI density. In 2011-2014 were 1216, 680, 1096 and 814 reviews respectively, 85.5%, 88.5%, 89.5% and 88.6% compliance actions. In relation to EPI density enters these years were: 0.85; 2.81; 1.02 and 0 (zero) in the year 2014. In evaluating the past eighteen months (July 2013 to December 2014), we found that there was no case of pneumonia associated with mechanical ventilation, a fact that motivated the drive to celebrate importance of working together.

## Conclusion

The interdisciplinarity of the sectors involved in the activities highlighted the importance of teamwork in order to better results in processes and quality of care.

## Disclosure of interest

None declared.

